# Biobtree: A tool to search and map bioinformatics identifiers and special keywords

**DOI:** 10.12688/f1000research.17927.4

**Published:** 2020-01-20

**Authors:** Tamer Gur

**Affiliations:** 1 European Bioinformatics Institute, Wellcome Trust Genome Campus, Hinxton, CB10 1SD Cambridge, UK

**Keywords:** bioinformatics, identifiers, search, mapping, visualization

## Abstract

Biobtree is a bioinformatics tool to search and map bioinformatics datasets via identifiers or special keywords such as species name. It processes large bioinformatics datasets using a specialized MapReduce-based solution with optimum computational and storage resource usage. It provides uniform and B+ tree-based database output, a web interface, web services and allows performing chain mapping queries between datasets. It can be used via a single executable file or alternatively it can be used via the R or Python-based wrapper packages which are additionally provided for easier integration into existing pipelines. Biobtree is open source and available at GitHub.

## Introduction

 Mapping bioinformatics datasets through a web interface or programmatically via identifiers or special keywords and attributes such as gene name, gene location, protein accessions and species name is a common need during genomics research. These mappings play an essential role in molecular data integration ( Huang *et al.*, 2011) and allow the gathering of maximum biological insight ( Mudunuri *et al.*, 2009) for these diverse bioinformatics datasets.

There are several existing tools for these mapping needs; these tools are gene-centric, protein-centric or can provide both gene- and protein-centric solutions. One of the common gene-centric tools is BioMart ( Zhang *et al.*, 2011)-based tools such as Ensembl BioMarts ( Kinsella *et al.*, 2011) which covers Ensembl ( Zerbino *et al.*, 2018) and Ensembl Genomes ( Kersey *et al.*, 2018) datasets. The R programming language package biomarRt ( Durinck *et al.*, 2009) is also widely used via performing queries with BioMart-based tools. Other common gene-centric tools are MyGene.info ( Xin *et al.*, 2016), DAVID ( Huang da *et al.*, 2009) and g:Profiler ( Raudvere *et al.*, 2019). Uniprot ID mapping service ( Huang *et al.*, 2011) provides a protein-centric solution. bioDBnet ( Mudunuri *et al.*, 2009) and BridgeDb ( van Iersel *et al.*, 2010) provide services for both gene- and protein-centric solutions.

On the other hand, genomics data size is increasing continuously ( Langmead & Nellore, 2018) especially via high throughput sequencing, so performing these mappings on these expanding data sizes in local computers, cloud computing or existing computing environments in a rapid and effective way via tools with easy installation and requiring minimum maintenance is a challenge ( Marx, 2013).

The referenced existing gene-centric tools currently do not support large Ensembl Bacteria genomes. Existing tools either provide only online services or require specific technical knowledge such as a particular database or specific programming language to install, use and adapt to different computational environments such as a local computer. Another limitation of the referenced tools is that they provide one-dimensional filtering capability in a single mapping query.

Biobtree addresses these problems of existing tools, First, it can be used via a single executable file without requiring re-compilation or extra maintenance such as database administration. Alternatively, it can be used via the R or Python-based wrapper packages which have been provided to allow for easier integration into existing pipelines. To process large datasets, it uses a specialized MapReduce-based solution which is discussed in the next section. MapReduce is an effective way to deal with large datasets ( Langmead & Nellore, 2018). After processing data, Biobtree provides a web interface, web services and chain mapping and filtering query capability in a single query with its intuitive query syntax which is demonstrated in the use cases section. Biobtree covers a range of bioinformatics datasets including Ensembl Bacteria genomes. The data resources currently used are ChEBI ( Hastings *et al.*, 2016), HGNC ( Braschi *et al.*, 2019), HMDB ( Wishart *et al.*, 2018), InterPro ( Mitchell *et al.*, 2019), Europe PMC ( Europe PMC Consortium, 2015), UniProt ( UniProt Consortium, 2019), Chembl ( Gaulton *et al.*, 2017), Gene Ontology ( The Gene Ontology Consortium, 2019), EFO ( Malone *et al.*, 2010), ECO ( Giglio *et al.*, 2019), Ensembl ( Zerbino *et al.*, 2018) and Ensembl Genomes ( Kersey *et al.*, 2018). Table 1 shows details of these datasets.

**Table 1.** List of datasets.

Dataset	Description	Location	Format
ChEBI	ChEBI reference accession data	ftp.ebi.ac.uk/chebi/Flat_file_tab_delimited/	TSV
HGNC	Human gene nomenclature	ftp.ebi.ac.uk/genenames/new/json/	JSON
HMDB	Human metabolome database	http://www.hmdb.ca/system/downloads/current/	XML
InterPro	Protein Families	ftp://ftp.ebi.ac.uk/pub/databases/interpro/current	XML
Literature mappings	Literature pmid, pmcid and doi mappings	ftp://ftp.ebi.ac.uk/pub/databases/pmc/DOI/	CSV
Taxonomy	NCBI Taxonomy	ftp://ftp.ebi.ac.uk/pub/databases/taxonomy/	XML
Uniparc	UniProt Sequence Archive	ftp.ebi.ac.uk/pub/databases/uniprot/current_release/uniparc/	XML
UniProt reviewed	UniProt Knowledgebase reviewed	ftp.ebi.ac.uk/pub/databases/uniprot/current_release/knowledgebase/complete/	XML
UniProt unreviewed	UniProt Knowledgebase unreviewed	ftp.ebi.ac.uk/pub/databases/uniprot/current_release/knowledgebase/complete/	XML
Uniref50	UniProt sequence clusters	ftp.ebi.ac.uk/pub/databases/uniprot/current_release/uniref/uniref50/	XML
Uniref90	UniProt sequence clusters	ftp.ebi.ac.uk/pub/databases/uniprot/current_release/uniref/uniref90/	XML
Uniref100	UniProt sequence clusters	ftp.ebi.ac.uk/pub/databases/uniprot/current_release/uniref/uniref100/	XML
GO	Gene Ontology	http://purl.obolibrary.org/obo/go.owl	RDF/XML
ECO	The Evidence & Conclusion Ontology	http://purl.obolibrary.org/obo/eco.owl	RDF/XML
EFO	Experimental Factor Ontology	http://www.ebi.ac.uk/efo/efo.owl	RDF/XML
ChEMBL	Chemical database of bioactive molecules	ftp.ebi.ac.uk/pub/databases/chembl/ChEMBL-RDF/latest/	RDF/XML
Ensembl	Ensembl	ftp.ensembl.org/pub/current_json/ ftp.ensembl.org/pub/current_mysql/ ftp.ensembl.org/pub/current_gff3/	JSON,CSV, GFF3
Ensembl Genomes Metazoa	Ensembl Genomes Metazoa	ftp://ftp.ensemblgenomes.org/pub/current/metazoa/json/ ftp://ftp.ensemblgenomes.org/pub/current/metazoa/mysql/ ftp://ftp.ensemblgenomes.org/pub/current/metazoa/gff3/	JSON,CSV, GFF3
Ensembl Genomes Plants	Ensembl Genomes Plants	ftp://ftp.ensemblgenomes.org/pub/current/plants/json/ ftp://ftp.ensemblgenomes.org/pub/current/plants/mysql/ ftp://ftp.ensemblgenomes.org/pub/current/plants/gff3/	JSON,CSV, GFF3
Ensembl Genomes Fungi	Ensembl Genomes Fungi	ftp://ftp.ensemblgenomes.org/pub/current/fungi/json/ ftp://ftp.ensemblgenomes.org/pub/current/fungi/mysql/ ftp://ftp.ensemblgenomes.org/pub/current/fungi/gff3/	JSON,CSV, GFF3
Ensembl Genomes Protists	Ensembl Genomes Protists	ftp://ftp.ensemblgenomes.org/pub/current/protists/json/ ftp://ftp.ensemblgenomes.org/pub/current/protists/mysql/ ftp://ftp.ensemblgenomes.org/pub/current/protists/gff3/	JSON,CSV, GFF3
Ensembl Genomes Bacteria	Ensembl Genomes Bacteria	ftp://ftp.ensemblgenomes.org/pub/current/bacteria/json/ ftp://ftp.ensemblgenomes.org/pub/current/bacteria/gff3/	JSON,GFF3

## Methods

### Implementation

The Biobtree implementation process starts by retrieving selected datasets as shown in Table 1 and retrieving data entries belonging to these datasets with their attributes and mapping information from their public locations, which are also shown in Table 1. During this data retrieval, the whole of the data do not get stored and uncompressed on the disk, instead data are retrieved and uncompressed in a streaming manner in the memory, which allows avoiding excessive disk space usage. Necessary data, which are these mapping and attributes, are compactly stored as chunks on the disk. During these data retrievals, all the idle CPU cores have been utilized to merge and sort these chunks recursively with each other. It is essential that the produced files are sorted to make fast batch inserts to the LMDB database which Biobtree uses as a database to store its result data. Once the data retrieval process is completed, result chunk files are globally merged using the patience sort technique and inserted into the LMDB database as keys and values. Keys consist of identifiers and special keywords such as gene names or species name, and values are attributes such as genomic coordinates and mapped identifiers. In these processes, data retrieval and creation of sorted chunks represent the map phase, global merge of the chunks and database creation represent the reduce phase of the MapReduce solution. Once the database is created, the Biobtree web module provides a web interface and web services to perform both searching for identifiers and mapping queries. Mapping queries has been done with a query syntax which allows chains of mapping and filtering between datasets. An example use case with this syntax is demonstrated in the next section. Biobtree uses a B+ tree data structure-based LMDB key-value store. LMDB provides fast batch inserts and reads which fits the bioinformatics datasets update cycle well where datasets are often updated periodically, and then only intensive read operations are performed. LMDB is embedded into Biobtree’s executable binary code so it does not require a separate installation or special maintenance.

## Use cases

The Biobtree web interface can be used primarily for exploration purposes and web services related to integrating genomic analysis pipelines via the Biobtree executable file which is available at GitHub. However, use of the Biobtree executable requires some learning of Biobtree usage and also, in relation to integrating into pipelines written in the various different languages, requires some extra coding effort.

In order to address the above situations, R and Python based wrapper packages BiobtreeR and BiobtreePy have been provided. R and Python are commonly used in genomic analysis ( Russell *et al.*, 2018). Both the BiobtreeR and BiobtreePy packages provide very similar functionalities and allow Biobtree to be used seamlessly within pipelines written in these languages.

The BiobtreeR package is provided via Bioconductor ( Huber *et al.*, 2015) and meets the build, test and quality standards stipulated for a Bioconductor package.

Another usability feature aimed at easier integration with existing pipelines, which was suggested in the course of the BiobtreeR package review process and has been implemented, is that of providing built-in Biobtree databases for commonly studied datasets and organism genomes. This feature is intended to speed up the data build and update processes related to common datasets and organism genomes and includes example use cases via the web interface. These latter serve the purpose of familiarizing users with the Biobtree and its query syntax.

The following three use cases are demonstrated using BiobtreeR and involve its installation instructions. The first two use cases employ built-in databases, but in the last use case data is built from genomes belonging to specific taxonomy identifiers which are not included in the built-in databases.

**BiobtreeR installation**


# install package                                                                       
if (!requireNamespace("BiocManager", quietly = TRUE))                                   
install.packages("BiocManager")                                                         
BiocManager::install("tamerh/biobtreeR")                                                

# import package                                                                        
library(biobtreeR)                                                                      

# set a output directory for biobtree files                                             
bbUseOutDir('set directory path')                                                       

# retrive built-in biobtree database for commonly studied dataset and organism genomes  
bbBuiltInDB()                                                                           

# start biobtree server                                                                 
bbStart()                                                                               


**usecase-1 Map Affymetrix identifiers to Ensembl human genome identifiers and then map these to the molecular function type GO term** **s**


# Given comma seperated Affymetrix identifiers maps to GO identifiers                                                                                          
bbMapping("202763_at,209310_s_at",'map(transcript).map(ensembl).map(go).filter(go.type=="molecular_function")',source = "affy_hg_u133_plus_2",attrs = "name")  

# query results                                                                                                                                                
##          input       input_dataset mapping_id                                                                                                               
## 1    202763_AT affy_hg_u133_plus_2 GO:0002020                                                                                                               
## 2            -                   - GO:0004190                                                                                                               
## 3            -                   - GO:0004197                                                                                                               
## 4            -                   - GO:0004861                                                                                                               
## 5            -                   - GO:0005123                                                                                                               
## 6            -                   - GO:0005515                                                                                                               
## 7            -                   - GO:0008233                                                                                                               
## 8            -                   - GO:0008234                                                                                                               
## 9            -                   - GO:0016005                                                                                                               
## 10           -                   - GO:0016787                                                                                                               
## 11           -                   - GO:0044877                                                                                                               
## 12           -                   - GO:0097153                                                                                                               
## 13           -                   - GO:0097199                                                                                                               
## 14           -                   - GO:0097200                                                                                                               
## 15 209310_S_AT affy_hg_u133_plus_2 GO:0004197                                                                                                               
## 16           -                   - GO:0005515                                                                                                               
## 17           -                   - GO:0008233                                                                                                               
## 18           -                   - GO:0008234                                                                                                               
## 19           -                   - GO:0016787                                                                                                               
## 20           -                   - GO:0050700                                                                                                               
## 21           -                   - GO:0097199                                                                                                               
##                                                                             name                                                                            
## 1                                                               protease binding                                                                            
## 2                                           aspartic-type endopeptidase activity                                                                            
## 3                                           cysteine-type endopeptidase activity                                                                            
## 4            cyclin-dependent protein serine/threonine kinase inhibitor activity                                                                            
## 5                                                         death receptor binding                                                                            
## 6                                                                protein binding                                                                            
## 7                                                             peptidase activity                                                                            
## 8                                               cysteine-type peptidase activity                                                                            
## 9                                            phospholipase A2 activator activity                                                                            
## 10                                                            hydrolase activity                                                                            
## 11                                            protein-containing complex binding                                                                            
## 12            cysteine-type endopeptidase activity involved in apoptotic process                                                                            
## 13  cysteine-type endopeptidase activity involved in apoptotic signaling pathway                                                                            
## 14 cysteine-type endopeptidase activity involved in execution phase of apoptosis                                                                            
## 15                                          cysteine-type endopeptidase activity                                                                            
## 16                                                               protein binding                                                                            
## 17                                                            peptidase activity                                                                            
## 18                                              cysteine-type peptidase activity                                                                            
## 19                                                            hydrolase activity                                                                            
## 20                                                           CARD domain binding                                                                            
## 21  cysteine-type endopeptidase activity involved in apoptotic signaling pathway                                                                            


**usecase-2 Map human Ensembl identifiers with given genome location to the reviewed Uniprot identifiers**


# 'homo sapiens' refers to identifier of 9606 in taxonomy                                                                                                                
# built-in 'within' genomic range function in the query which                                                                                                            
# equivalents to ensembl.start>100000000 && ensembl.end< 101000000                                                                                                       
bbMapping('homo sapiens','map(ensembl).filter(ensembl.within(100000000,101000000) && ensembl.seq_region=="X").map(uniprot).filter(uniprot.reviewed)',attrs = "names[1]" )

# query results                                                                                                                                                          
##   mapping_id                                 names[1]                                                                                                                 
## 1     O43657                            Tetraspanin-6                                                                                                                 
## 2     Q9H2S6                              Tenomodulin                                                                                                                 
## 3     Q9Y5S8                          NADPH oxidase 1                                                                                                                 
## 4     P33240    Cleavage stimulation factor subunit 2                                                                                                                 
## 5     O60687    Sushi repeat-containing protein SRPX2                                                                                                                 
## 6     Q96C24             Synaptotagmin-like protein 4                                                                                                                 
## 7     Q8TAB3                         Protocadherin-19                                                                                                                 
## 8     Q5H913 ADP-ribosylation factor-like protein 13A                                                                                                                 
## 9     Q6PP77                     XK-related protein 2                                                                                                                 


**usecase-3 Map all taxonomic children of given bacteria and then map these children to Ensembl with given genome location and contains a given word**


# this use case requires new data build                                                                                                     
# stop running server                                                                                                                       
# clean output directory or set new one to keep both data                                                                                   
bbStop()                                                                                                                                    

# build data with specific bacteria genomes                                                                                                 
bbBuildCustomDB(taxonomyIDs = "595,984254,465517,1249525")                                                                                  

# start server with new data                                                                                                                
bbStart()                                                                                                                                   

# taxonomy identifier 59201 is used instead of full name 'Salmonella enterica subsp. enterica'                                              
bbMapping("59201",'map(taxchild).map(ensembl).filter(ensembl.start<10000&&ensembl.description.contains("SopD"))',attrs="strand,start,end")  

# query results                                                                                                                             
##      mapping_id strand start   end                                                                                                       
## 1   ACH54_23895      +  2525  3484                                                                                                       
## 2   ACH56_04205      -    27   986                                                                                                       
## 3   AEW14_05145      -  3410  4369                                                                                                       
## 4   AEW14_15935      -     1    89                                                                                                       
## 5    DE27_21250      +  8885  9967                                                                                                       
## 6    DE87_06330      +  7839  8921                                                                                                       
## 7 LPMST02_21800      +  8983 10065                                                                                                       


## Discussion

A mapping between bioinformatics datasets via identifiers or special keywords such as species names is often performed during genomic analyses and plays an essential role in molecular data integration and getting maximum biological insight from these datasets. There are several gene-centric, protein-centric and both protein- and gene-centric tools for addressing these mapping needs. These tools currently do not support the large Ensembl Genomes Bacteria dataset. In addition, these tools provide either only online services or require specific technical knowledge to install and adapt to new computing environments. Existing tools also provide one-dimensional filtering in a single mapping query. Biobtree addresses these problems by managing a tool with a single executable file or alternatively additionally provided R or Python based wrapper packages and processing large datasets with its specialized MapReduce-based solution. Based on processed data, it creates a uniform database and allows searching identifiers and chain mappings and filtering queries.

## Data availability

All data underlying the results are available as part of the article and no additional source data are required.

## Software availability

**All source codes and binaries available at:** https://www.github.com/tamerh/biobtree.

**Archived source code at time of publication:** https://doi.org/10.5281/zenodo.2547047

**License:** BSD 3-Clause “New” or “Revised” license.
