# Supplementary material for: Biobtree: A tool to search and map bioinformatics identifiers and special keywords
Source: F1000Res. 2020 Jan 20;8:ISCB Comm J-145. Originally published 2019 Feb 4. [Version 4] doi: 10.12688/f1000research.17927.4 (PMC6964641; doi:10.12688/f1000research.17927.4)
Supplement: Peer review history

# Peer review: Reviewer response for version 4

**DOI:** 10.5256/f1000research.24382.r58843

**Published:** 2020-01-21

**Authors:** Samuel Lampa

**Affiliations:** 1 Department of Pharmaceutical Biosciences, Uppsala University, Uppsala, Sweden; 2 Savantic AB, Stockholm, Sweden

I hereby again confirm my approval status. Great work!

I confirm that I have read this submission and believe that I have an appropriate level of expertise to confirm that it is of an acceptable scientific standard.

# Peer review: Reviewer response for version 3

**DOI:** 10.5256/f1000research.24176.r58289

**Published:** 2020-01-15

**Authors:** Maxim N. Shokhirev

**Affiliations:** 1 Razavi Newman Integrative Genomics and Bioinformatics Core, Salk Institute for Biological Studies, La Jolla, CA, USA

The author has made additional efforts to improve the installation and use of the tool. I am happy to accept it and hope the author will continue to improve and maintain it over time.

I confirm that I have read this submission and believe that I have an appropriate level of expertise to confirm that it is of an acceptable scientific standard.

# Response

**Published:** 2020-01-16

**Authors:** Tamer Gur

**Affiliations:** EMBL European Bioinformatics Institute, UK

Thank you for your review and suggestions to make the article accepted. I have submitted a new version of the article for the English language issues. And yes I will maintain and improve the tool. Especially maintenance is necessary for the Bioconductor package. I will also add major new features if I get requests from users.

# Peer review: Reviewer response for version 3

**DOI:** 10.5256/f1000research.24176.r58288

**Published:** 2020-01-13

**Authors:** Samuel Lampa

**Affiliations:** 1 Department of Pharmaceutical Biosciences, Uppsala University, Uppsala, Sweden; 2 Savantic AB, Stockholm, Sweden

I hereby confirm my approval status of this article, while suggesting to fix the following language issues:

**Introduction:**

- "performing these mapping" --> "performing these mappings".
- "provide only online service" --> "provide only online services".
- "all the idle CPUs" --> "all the idle CPU cores".
  (This is also to be more technically correct, as few computers these days have multiple CPUs, while most of them have CPUs with multiple cores).

I confirm that I have read this submission and believe that I have an appropriate level of expertise to confirm that it is of an acceptable scientific standard.

# Response

**Published:** 2020-01-16

**Authors:** Tamer Gur

**Affiliations:** EMBL European Bioinformatics Institute, UK

Thank you again for your review and suggestions to make the article accepted.

I have submitted the new version for the English language issues.

# Peer review: Reviewer response for version 2

**DOI:** 10.5256/f1000research.22620.r53983

**Published:** 2019-10-21

**Authors:** Samuel Lampa

**Affiliations:** 1 Department of Pharmaceutical Biosciences, Uppsala University, Uppsala, Sweden; 2 Savantic AB, Stockholm, Sweden

The revised article is now much improved, with a solid introduction explaining the problem area and previous research, use cases, and with the title amended to better reflect the functionality of the tool. I think it can now be approved, with the following minor suggestions for language fixes:

**Abstract**

 web interface -> a web interface

**Introduction**

 P. 3: Biobtree address -> Biobtree addresses

**Methods**

 I think "disc" should be "disk" for hard drive disks.

 as key and values -> as keys and values

 "... and values are attributes and mapped datasets information".

 This is a somewhat unclear sentence. I.e, does it mean "mapped datasets' information" (genitive), or "information about mapped datasets"? I suggest slightly clarifying it.

**Use cases**

 "... third input is dataset to" -> "... third input is a dataset to"

 "in different dataset" -> "different datasets" or "a different dataset"

 "with same value" -> "with the same value"

**Future work**

 "In addition, following and experimenting with the advancements in large data processing techniques, databases and data structures fields to improve the tool further".

 This sentence seems incomplete. I.e, what is stated about this activity? Something that is planned to be done, or "could be done in general"?

I confirm that I have read this submission and believe that I have an appropriate level of expertise to confirm that it is of an acceptable scientific standard.

# Response

**Published:** 2019-10-22

**Authors:** Tamer Gur

**Affiliations:** EMBL European Bioinformatics Institute, UK

Thank you very much for taking time to review the article and for all the comments and suggestions. I will address your new minor suggestions in the next version together with demonstrating the use cases with R package.

# Peer review: Reviewer response for version 2

**DOI:** 10.5256/f1000research.22620.r53984

**Published:** 2019-09-27

**Authors:** Maxim N. Shokhirev

**Affiliations:** 1 Razavi Newman Integrative Genomics and Bioinformatics Core, Salk Institute for Biological Studies, La Jolla, CA, USA

The author now includes references to previously published annotation tools and services such as BioMart and MyGene.info and points out several limitations of the extant tools that are addressed with Biobtree. In addition, the author now includes an online portal for exploring data with a structured query language along with a few examples of specific use cases and a table of databases implemented.

 Given the improvements, the work can now be beneficial for biologists interested in mapping and annotation of specific genes or terms, which can serve as an alternative to already established annotation databases and services.

 However, there are still several limitations to this work. First, supposedly one of the main advantages of Biobtree is that it can be run as a standalone executable tool. This is great in theory but there doesn't seem to be any executables in the referenced repository or an explanation of how to install the Biobtree in various environments. The manuscript goes into some detail about the underlying algorithms and datastructures, but there is no explanation of what is required for you to install the program or get it working in either the manuscript or the repository. Therefore, without documentation or description the only way to use the tool currently is through the online portal. The second major limitation is that while there are three use-cases described, there is no documentation provided for how one would build arbitrary queries. A manual or easier to use interface is needed before the full power of the tool can be leveraged. Finally, I couldn't find a way to download the results from the online portal. There is often hundreds of pages of results but it seems there is no way to obtain a table of the results for use in downstream tools or analyses. Given the fact that there is no description of how to install the tool or which prerequisites are needed, and the fact that you can't download the results from the online portal, this tool is currently only useful for manual exploration of the mappings and not suitable for inclusion in bioinformatics pipelines. Since it seems the author intended for the latter, instructions for how to install the tool, a detailed description of the API or commands, and a way to save the output to a table of some kind is still needed. I look forward to seeing the revised manuscript.

I confirm that I have read this submission and believe that I have an appropriate level of expertise to confirm that it is of an acceptable scientific standard, however I have significant reservations, as outlined above.

# Peer review: Reviewer response for version 1

**DOI:** 10.5256/f1000research.19605.r46335

**Published:** 2019-04-15

**Authors:** Samuel Lampa

**Affiliations:** 1 Department of Pharmaceutical Biosciences, Uppsala University, Uppsala, Sweden; 2 Savantic AB, Stockholm, Sweden

The article describes a commandline tool, Biobtree, that is claimed to allow to process relations between bioinformatics datasets based on various characteristics such as identifiers and keywords.

 The manuscript describes the tool in a clear way technically, making it quite clear what it does in technical terms, and how it is supposed to be used.

 Also, I was able to install and run the tool in a simple way on my laptop (i5 CPU, 8GB RAM and 10-15 GB free hard drive, Xubuntu 16.04 64 bit) without problems. It provides a simple but good looking and easy to use web interface.

 I'm seeing at least two major issues with the tool and manuscript though, that needs being thoroughly addressed to make them acceptable.

**Main problem 1: Visualization?**

 Firstly, the title claims that the tool does visualization of the database produced by the tool. Perhaps I'm missing something, but I have not found any visualization in the tool apart from a form of search hit result listings. I don't think this is enough to be called "visualization". Especially as it is unclear how the current form of output is supposed to be used in a concrete biological usecase. With the current wording, I would expect something more graphical, like a graphviz-like graph view of dataset relations.

 Suggested edits to make the tool and paper acceptable:

- Provide graphical visualization beyond results listings (or explain how to show them, if I have missed them), or else remove "visualization" from the title and other places.
- Use this/these visualizations in the use cases/demonstrators discussed above, to explain how they contribute to solving concrete biological problems.

**Main problem 2: Lack of context and discussion of biological relevance**

 The first and main problem with the manuscript is that it does not provide a clear enough description of what *biological* problem it is solving. Nor does it provide an overview of existing tools and solutions in this field. Right now, the manuscript only states what the tool can do in technical terms. It somehow reads like a (well written) user guide or README file, but not yet a scientific paper. To help potential new users understand why they might need this tool, it needs to be put in context and compared with other existing tools.

 In my view, the manuscript needs the following points thoroughly addressing to be acceptable:

1. In the introduction: Elaborate on the field of mapping/visualising dataset relations, mentioning relevant existing similar tools, what are the typical problems, and what particular problem Biobtree solves.
2. Explain a few examples of *biological* problems that can be solved with this tool, or type of tool.
3. E.g. in the results: Provide at least one, and optimally two or three potentially simple, but relevant, biological demonstrators or use cases, that can be addressed with the tool. Provide complete instructions on how to re-run this or these demo(s) and provide outputs for this/these in terms of figures or diagrams and how these were produced. In this way, both reviewers and users can make sure that they understand how to operate the tool.
4. In the discussion: Connect back to the explained problem the tool is addressing, and explain how the problem was solved, again reinstating the relevance of this specific tool compared to other existing tools, and what improvement it provides to the end user trying to solve biological problems, exemplified by the demonstrators or use cases.

**Language issues**

 The manuscript also contains quite a number of language issues. I'm listing a few language suggestions below as examples, but further language proofing or editing is highly recommended, to make sure there are not more of these:

1. **Methods** section:
  "in GO programming language" --> "in the Go programming language"
  (Note the "the" and that only G is uppercase in "Go").
2. **Update phase** section:
  "to LMDB" -> "to the LMDB"
3. **Update phase** section:
  "Updating reads selected datasets as a stream"
  I don't understand this sentence. Please language-check it.
4. "is used in next" -> "is used in the next"
5. **Generate phase** section:
  "the project github page" -> "the project's GitHub page"
6. **Web phase **section:
  "starts web phase" -> "starts the web phase"
7. **Web interface** section:
  "The Web interface allow user" -> "The web interface allows the user"
8. **Operation **section:
  "Biobtree executable" -> "The biobtree executable".

I confirm that I have read this submission and believe that I have an appropriate level of expertise to confirm that it is of an acceptable scientific standard, however I have significant reservations, as outlined above.

# Peer review: Reviewer response for version 1

**DOI:** 10.5256/f1000research.19605.r45074

**Published:** 2019-03-07

**Authors:** Maxim N. Shokhirev

**Affiliations:** 1 Razavi Newman Integrative Genomics and Bioinformatics Core, Salk Institute for Biological Studies, La Jolla, CA, USA

While it is important to create a consistent and queryable database of biological identifiers, it is unclear what advances this tool brings to the field. For example, how does this tool compare to other queryable database tools such as mygene.info, or BioMart? The paper will greatly benefit from a comparison to these and other such tools.

 I downloaded and ran the tool but it seems I can't get through the update phase when I run ./biobtree update (It seems to hang after uniprot_reviewed finishes) without any other messages. When I rerun using biobtree --d uniprot_reviewed update it finishes but there is an error: 

 Error while reading file-> .//out/index/0_13.938476000.gz

 panic: gzip: invalid header

 I tried running generate and web after that regardless, but couldn't get it to work:

 panic: mdb_txn_commit: MDB_BAD_TXN: Transaction must abort, has a child, or is invalid

 Error while reading meta information file which should be produced with generate command. Please make sure you did previous steps correctly.

 The author needs to debug/test their code to ensure that it can be used by others.

I confirm that I have read this submission and believe that I have an appropriate level of expertise to state that I do not consider it to be of an acceptable scientific standard, for reasons outlined above.

# Response

**Published:** 2019-03-10

**Authors:** Tamer Gur

**Affiliations:** EMBL European Bioinformatics Institute, UK

Thank you for reviewing the article. I agree that there are several similar tools exist with different dataset and functionalities such as Biomart and mygene.info. However, this tool can still complement them for following main reasons.

- Biobtree can work in local machine. This can be especially useful when large number of requests needs to be performed. For instance currently similar Uniprot tool documentation suggests either split the requests or download underlying data when number of requests are above 50K. These types of limitations for bulk requests are sensible for fair usage of a public service and can be more suitable with Biobtree type locally runnable tool.
- Users custom dataset can be integrated. 
- Tool provides new intuitive web interface.

In relation to reported errors, I have added demo of tool in case such errors happen again. I have also added integration test which runs periodically on Linux, MacOS and Windows operating systems via Azure DevOps platform. These tests can be accessed publicly and test and demo links can be found at github page. Based on these tests, it seems that tool is working as expected. I believe that hanged process is a specific issue or bug which I am happy to resolve if I have more information. The rest of problems which have been reported are most probably due to the prematurely exited hanged process. Either starting in a new folder or passing --clean parameter can solve the issue.
